# Evaluation of the reproducibility of facial profile photographic records taken in two different head positions

**DOI:** 10.1590/2177-6709.30.1.e2524235.oar

**Published:** 2025-03-24

**Authors:** Zenayde Godinho MARIANO, Ertty SILVA, Sérgio Carmelo TÔRRES, An TIEN-LI, Fernanda MELOTI, Irene MENDEZ-MANJON, Maurício de Almeida CARDOSO

**Affiliations:** 1 São Leopoldo Mandic College, School of Dentistry, Department of Orthodontics (Campinas/SP, Brazil).; 2 Institute of Health Studies - IES, School of Dentistry, Department of Orthodontics (Belo Horizonte/MG, Brazil).; 3 University of Brasília, Faculty of Health Sciences, Department of Periodontics (Brasília/DF, Brazil).; 4 International University of Catalonia, Faculty of Dentistry, Department of Oral and Maxillofacial Surgery (Sant Cugat del Vallès, Barcelona, Spain).

**Keywords:** Photography, Natural head position, Oriented position, Fotografia, Posição natural de cabeça, Posição orientada

## Abstract

**Objective:**

This study aimed to evaluate the intra- and inter-examiner reproducibility of photographic records of the facial profile in Natural Head Position (NHP) and Oriented Position (OP) taken by four examiners (two orthodontists, one oral and maxillofacial surgeon, and one photography technician).

**Methods:**

Each professional captured two lateral photographs of 25 individuals during the first (T1) and second phases (T2), to assess the reproducibility of the technique and method error using angular measurements, based on the soft tissue Glabella and soft tissue Pogonion landmarks. The Shapiro-Wilk normality test and intraclass correlation coefficient (ICC) tests, using a two-way mixed model, were applied. To assess the agreement between the NHP and OP methods, Bland-Altman analysis was conducted, followed by regression analysis. The significance level for all statistical analyses was set at 5%.

**Results:**

The ICC analysis for the angular measurement in the NHP position demonstrated good inter-examiner reliability at T1 (ICC=0.706; p<0.001) and T2 (ICC=0.739; p<0.001). In the intra-examiner analysis, only examiner 1 (orthodontist) showed moderate reliability (ICC=0.504; p<0.001). For the OP position, the analysis revealed good inter-examiner reliability at T1 (ICC=0.708; p<0.001) and T2 (ICC=0.704; p<0.001). Again, only examiner 1 (orthodontist) exhibited moderate intra-examiner reliability (ICC=0.530; p=0.033). The Bland-Altman plots demonstrated agreement between NHP and OP for each examiner at T1 and T2.

**Conclusion:**

The NHP and OP techniques were reliable when performed by technically proficient and calibrated professionals.

## RESUMO

**Objetivo:**

Este estudo teve como objetivo avaliar a reprodutibilidade intra e interexaminadores nos registros fotográficos do perfil facial em Posição Natural de Cabeça (PNC) e Posição Orientada (PO) realizadas por quatro examinadores (dois ortodontistas, um cirurgião bucomaxilofacial e um técnico em fotografia).

**Métodos:**

Cada profissional realizou duas fotografias laterais de 25 indivíduos, na primeira (T1) e segunda fases (T2) buscando avaliar a reprodutibilidade da técnica e o erro do método por meio de medidas angulares, utilizando os pontos glabela e pogônio mole. Aplicou-se o teste de normalidade de Shapiro-Wilk e os testes de correlação intraclasse (ICC), modelo misto de duas vias. Para avaliar a concordância entre os métodos PNC e PO, realizou-se a análise de Bland-Altman com posterior análise de regressão. O nível de significância para todas as análises estatísticas foi de 5%.

**Resultados:**

A análise de ICC para a medida angular para técnica PNC mostrou uma boa confiabilidade interexaminadores em T1 (ICC=0,706; p<0,001) e T2 (ICC=0,739; p<0,001). Na concordância intraexaminadores, verificou-se que apenas para o examinador 1 (ortodontista) houve uma confiabilidade razoável (ICC=0,504; p<0,001). Para a técnica PO, a análise mostrou uma boa confiabilidade interexaminadores em T1 (ICC=0,708 p<0,001) e T2 (ICC= 0,704; p<0,001). Verificou-se que apenas para o examinador 1 (ortodontista) foi constatada uma confiabilidade razoável (ICC=0,530; p= 0,033) intraexaminador. Os gráficos da análise de Bland-Altman mostraram concordância entre PNC e PO para cada examinador em T1 e T2.

**Conclusão:**

As técnicas PNC e PO mostraram-se confiáveis quando realizadas por profissionais com conhecimento técnico e calibrados.

## INTRODUCTION

The continuous search for harmony and balance in facial features is crucial in the contemporary concept of diagnosis and in the preparation of treatment plans.^1^ Clinical photographs are widely used in facial analysis, supporting diagnosis, planning, monitoring, and comparing the final results of orthodontic and surgical treatments.^2^

Digital photographs have been shown to be a simple, low-cost, non-invasive method with immediate visualization.^3^ ^,^ ^2^ When taking photographic records for clinical documentation, it is necessary to obtain comparable, accurate, and reproducible images.^4^ Standardization and positioning of the patient are relevant, allowing different photographers to continue a series of photographs of the same patient, as well as between different patients with the same condition.^5^

One of the limitations in facial analysis when using photographs is related to the position of the patient’s head. Head deviations or inclinations especially alter the sagittal position of the mandible^3^ ^,^ ^6^ so that every 1° of angulation alters the sagittal position of the mentum by 2mm.^6^ The orientation of the head influences the anteroposterior perception of the maxillomandibular complex and can result in an incorrect diagnosis, especially for patients with Class III malocclusion.^7^

During the photographic recording of the facial profile, the orientation of the head alignment can be based on references such as the Natural Head Position (NHP) or the Oriented Position (OP). The NHP was defined by Bjerin^8^ in 1957 and has been described as the most suitable position for taking facial profile photographs. It is considered a standardized and reproducible position.^2^ ^,^ ^9^ ^-^ ^13^

The OP is a position in which the individual is standing upright, with the body and head relaxed and, before taking the photo, the dentist adjusts the head to a position that they consider to be more natural.^14^ Thus, the position of the head is guided by the operator at the time of taking the photograph of the facial profile^7^ and is obtained through clinical training, presenting itself as an estimated and individual position.^7^ ^,^ ^9^

Reproducibility is another extremely important factor and, within these guidelines, Peng and Cooke^10^ described clinical reproducibility in a longitudinal study, concluding that the NHP is a reproducible position. The importance of reproducibility has been studied and proven by several other authors.^2^ ^,^ ^7^ ^,^ ^12^ ^,^ ^14^ ^,^ ^15^

Therefor, the present study aimed to assess intra- and inter-examiner reproducibility in photographic records of the facial profile in NHP and OP. The results may provide relevant information about the best positioning to use in clinical routine, helping orthodontists and oral and maxillofacial surgeons to diagnose, define the best therapeutic approach, and follow up after treatment.

## MATERIAL AND METHODS

The initial sample included 30 adult individuals of both sexes, irrespective of facial type or skeletal relationship, nasal breathers, with no previous history of facial surgery,^16^ craniofacial trauma,^1^ absence of major facial asymmetries^1^ ^,^ ^17^ or congenital anomalies. The sample size was calculated in line with Lundström et al.^18^ After evaluating all the photographic records, five individuals were excluded from the study because their lips were not relaxed. Thus, the final sample comprised 25 individuals. All of them signed an Informed Consent Form, and the study was approved by the Ethics Committee under number 5.055.798.

The photographs were taken by four professionals from different categories: two orthodontists, one oral and maxillofacial surgeon, and one photography technician. All had more than 15 years of clinical expertise in photography. The NHP and OP images were downloaded into computer folders after being taken by all four examiners during phase T1. Sixty days later, the NHP and OP photographs were retaken, to assess the reproducibility of the technique and the error of the method, and the images were again downloaded into computer folders. Subsequently, all the photographs were checked and at this point, it was found that during the photographic recording, five individuals did not have relaxed facial muscles, specifically the perioral region, while smiling. They were omitted from the study, since the muscles must be relaxed in order to obtain NHP, according to Solow and Tallgren’s procedure. All the photographic images were taken at the Faculdade de Medicina e Odontologia São Leopoldo Mandic (Campinas, São Paulo, Brazil).

The photographs were taken using two Canon EOS 6D Mark ll DSLR cameras and two Canon Macro 100mm lenses, which were selected to maintain the natural proportions of the subjects.^19^ The cameras were set up vertically on two Canon SL3600 tripods (1.80 m) to control stability and height. Two 50x70 cm softbox diffusers installed on tripods were used, positioned in front of the patient’s face at a distance of 1.5 meters. 

The positioning of the examiners and subjects was standardized using markings on the floor, to the side of the subject’s face. The cameras were calibrated and photographic tests were carried out beforehand, to check for possible image distortions. 

A 60x40 cm mirror was used, positioned two meters from the subject. A piece of graph paper (4x4 cm) was used on the background wall, at a distance of 50 cm from the subject’s face, in order to represent the true vertical and horizontal lines. In all the photographic records, the same distances were kept between the equipment, accessories, and the markings on the floor. Only the mirror was removed during the recordings in the OP. 

The position of the head during the recordings was determined in two ways: NHP^11^ and OP. In OP, the individual was instructed to maintain an ortho position posture, with natural head balance;^11^ the mirror was removed and then they were instructed to look at a point far away at eye level. At this point, the professional adjusted the individual’s head, when necessary, according to their perception, and then took a photograph. All individuals wearing glasses were asked to remove them.

All the images were downloaded into folders and organized on an ASUS Intel laptop for later review. The next phase consisted of marking the reference points on the photographs and obtaining the angular measurements. The points chosen were soft tissue glabella (G’) and soft tissue pogonion (SP’).^6^

The high-resolution images were stored in JPEG format and imported into the Angle Meter 360º app, installed on a 256GB OP 12.9-inch Apple iPad Pro Wi-Fi. The original file format was maintained, to avoid loss of image quality. The app was customized to provide black lines of minimum thickness, and demarcation of 4 points to allow two lines to be drawn: the G’-SP line and the true vertical. These points were marked with the smallest size, in black, and with the use of a magnifying glass to increase the precision of the marking of the anatomical points. 

**Figure 1:**
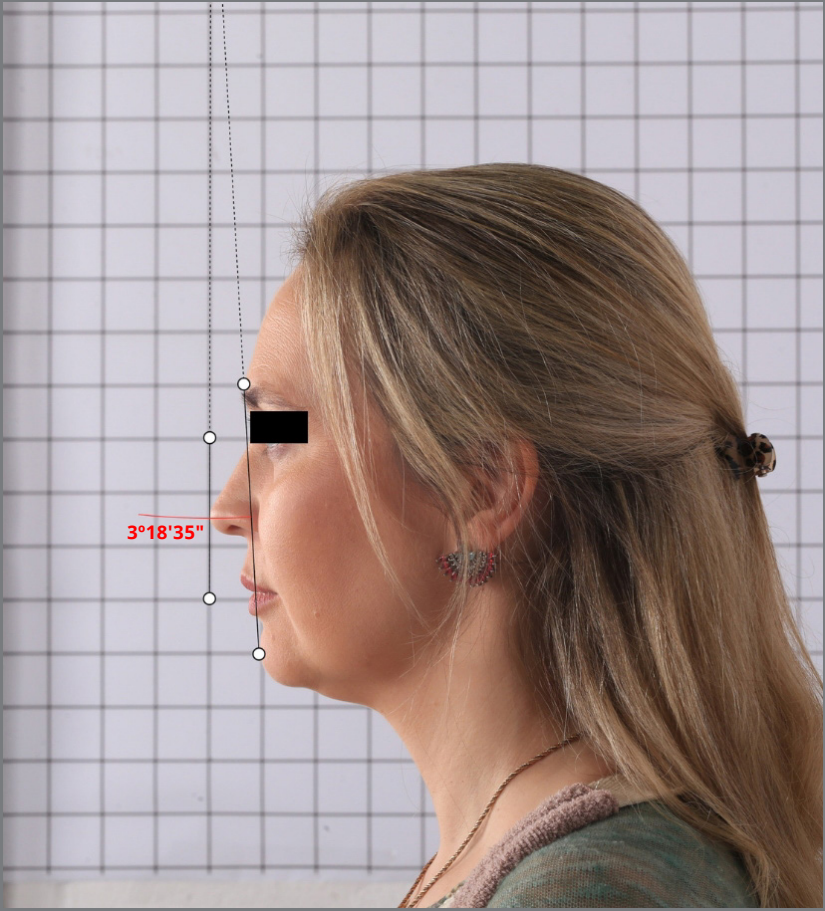
Measurement of the G’-SP’ line, the true vertical line, and the angle, using the Angle Meter 360º App (photograph taken of the sample at T2, examiner 2, in OP ).

The G’ and SP points were marked digitally on the device’s screen using an Apple Pencil, 2nd generation, magnetic connector (MU8F2AM/A), which increases the accuracy of the marking (Fig 1). The other two points were marked following one of the vertical lines on the grid background. The angular measurements were generated automatically using the Angle Meter 360º app. After obtaining the new images with the lines and angles, they were saved and archived again. 

In order to be submitted to statistical analysis, the data obtained was first tabulated, then the measurements were converted from degrees, minutes, and seconds into degrees (common units) using the Convert-me.com online application available at https://www.convert-me.com/pt/convert/circular/degminsec/degminsec-to-degree.html.

## STATISTICAL ANALYSIS

All the angular measurements were taken individually for each photographic record by the same examiner. Once collected, the measured values were organized and submitted to statistical treatment using Excel and SPSS v. 24.0 (IBM Corp., release 2016. IBM SPSS Statistics for Windows. Armonk, NY, EUA). The Shapiro-Wilk normality test was first applied to verify the distribution of the data. 

In order to calibrate the angle measurement process, a second measurement was carried out on the entire sample at T1 and T2, after 75 days, to calculate the method’s error. To detect random error, the formula recommended by Dahlberg was used: E^2^ = Σd^2^/2n.^20^ ^,^ ^21^ To detect systematic error, Student’s t-test was used for paired data, comparing the values obtained in the first and second measurements. 

Once the data had a normal distribution, they were subjected to intraclass correlation (ICC) tests, a two-way mixed model,^22^ to compare: (1) the reliability of the photographic records (NHP and OP) considering the professionals in T1 (intra and inter-examiner); (2) the reliability of the photographic records (NHP and OP) considering the professionals in T2 (intra and inter-examiner). In order to decide the degree of reliability, the following were considered: poor (ICC <0.40), reasonable (ICC between 0.40 and 0.59), good (ICC between 0.60 and 0.74), and excellent reliability (equal to or greater than 0.75).^23^

To assess the agreement between the NHP and OP methods, the Bland-Altman analysis^24^ was carried out, considering a significance level of 5% for all statistical analyses.

## RESULTS

Analysis of the comparison of angle measurements taken at T1 and T2 showed that there was no significant difference between the values measured at the two times, i.e. they showed minimal error and no significant random error, indicating that the researcher calibrated the measurement of angles in the photographs (Table 1). Comparing the mean angles obtained in measurements 1 and 2, it can be seen that they differ by less than 1 degree on average.

**Table 1:** Mean values of the angles of the first and second measurements in NHP and OP, considering the photographs produced by the four examiners and the respective values of the method error analysis using the Dahlberg test.

		M1 ± SD	M2 ± SD	Dahlberg	p value
NHP (T1)	E1	3.70 ± 2.38	3.63 ± 2.26	0.254	0.314
	E2	3.00 ± 2.04	2.82 ± 1.92	0.828	0.464
	E3	3.35 ± 2.55	3.37 ± 2.66	0.181	0.639
	E4	2.98 ± 2.07	3.02 ± 2.01	0.262	0.598
NHP (T2)	E1	3.46 ± 2.07	3.22 ± 2.11	0.439	0.059
	E2	3.02 ± 1.92	2.94 ± 1.74	0.442	0.548
	E3	2.95 ± 1.56	2.86 ± 1.74	0.356	0.347
	E4	3.06 ± 2.10	3.17 ± 2.19	0.532	0.460
OP (T1)	E1	3.75 ± 2.53	3.74 ± 2.50	0.221	0.920
	E2	2.78 ± 1.69	2.77 ± 1.80	0.261	0.920
	E3	3.34 ± 2.20	3.21 ± 2.27	0.671	0.509
	E4	3.03 ± 2.07	3.17 ± 2.11	0.274	0.083
OP (T2)	E1	3.21 ± 2.01	3.12 ± 2.03	0.186	0.082
	E2	2.87 ± 1.63	2.89 ± 1.68	0.308	0.795
	E3	3.16 ± 1.66	3.02 ± 1.64	0.275	0.065
	E4	3.22 ± 2.14	3.27 ± 2.14	0.205	0.415

M1: measurement 1; M2: measurement 2; T1: Time 1; T2: Time 2; E1: Examiner 1; E2: Examiner 2; E3: Examiner 3; E4: Examiner 4; NHP: Natural head position; OP: Oriented position.

The interclass ICC analysis for the OP angle measurement showed good inter-rater reliability in T1 (ICC = 0.706; 95% CI = 0.461-0.857; F = 3.404; p<0.001) (Table 2). Good inter-rater reliability was also observed in T2 (ICC = 0.739; 95% CI = 0.517-0.874; F = 3.747; p<0.001).

**Table 2:** Inter-examiner reliability for the value of the angles measured from the photographs taken in NHP in T1 and T2, assessed by the intraclass correlation coefficient (ICC), two-way mixed model.

Intraclass correlation			95% confidence interval		F-test with true value 0			
			Lower boundary	Upper boundary	Value	DF1	DF2	p
T1	Single measure	0.375	0.176	0.599	3.404	24	72	0.000
	Average measure	0.706	0.461	0.857	3.404	24	72	0.000
T2	Single measure	0.415	0.211	0.634	3.747	24	72	0.000
	Average measure	0.739	0.517	0.874	3.747	24	72	0.000

When assessing intra-examiner agreement for OP, reasonable reliability was observed only for examiner 1 (orthodontist) (ICC = 0.504; 95% CI = -0.106-0.78; F = 2.028; p<0.001) (Table 3).

**Table 3:** Intra-examiner reliability for angle measurements obtained from NHP photographs comparing T1 and T2, assessed by the two-way mixed model intraclass correlation coefficient (ICC).

Intraclass correlation			95% confidence interval		F-test with true value 0			
			Lower boundary	Upper boundary	Value	DF1	DF2	p
E1	Single measure	0.337	-0.050	0.639	2.028	24	24	0.045
	Average measure	0.504	-0.106	0.780	2.028	24	24	0.045
E2	Single measure	-0.149	-0.534	0.268	0.750	24	24	0.756
	Average measure	-0.350	-2.289	0.422	0.750	24	24	0.756
E3	Single measure	-0.204	-0.564	0.209	0.668	24	24	0.836
	Average measure	-0.512	-2.583	0.346	0.668	24	24	0.836
E4	Single measure	0.029	-0.383	0.421	1.058	24	24	0.445
	Average measure	0.057	-1.243	0.593	1.058	24	24	0.445

E1: Examiner 1; E2: Examiner 2; E3: Examiner 3; E4: Examiner 4; NHP: Natural head position; OP: Oriented position.

Good inter-rater reliability was also observed for the OP in T1 (ICC = 0.708; 95% CI = 0.467-0.858; F = 3.459; p<0.001) (Table 4). The same was observed when the inter-examiner data in T2 was analyzed (ICC = 0.704; 95% CI = 0.451-0.856; F = 3.307; p<0.001).

**Table 4:** Inter-examiner reliability for the angle measurements from the photographs taken in OP at T1 and T2, assessed by the two-way mixed model intraclass correlation coefficient (ICC).

Intraclass correlation		95% confidence interval			F-test with true value 0			
		Lower boundary		Upper boundary	Value	DF1	DF2	p
T1	Single measure	0.378	0.180	0.601	3.459	24	72	0.000
	Average measure	0.708	0.467	0.858	3.459	24	72	0.000
T2	Single measure	0.372	0.171	0.599	3.307	24	72	0.000
	Average measure	0.704	0.451	0.856	3.307	24	72	0.000

Reasonable reliability was found only for examiner 1 (orthodontist) when assessing intra-examiner agreement for OP (ICC = 0.530; 95% CI = -0.042-0.791; F = 2.151; p = 0.033) (Table 5).

**Table 5:** Intra-examiner reliability for angle measurements obtained in OP considering T1 and T2, assessed by the two-way mixed model intraclass correlation coefficient (ICC).

Intraclass correlation			95% confidence interval		F-test with true value 0			
			Lower boundary	Upper boundary	Value	DF1	DF2	P
E1	Single measure	0.361	-0.021	0.654	2.151	24	24	0.033
	Average measure	0.530	-0.042	0.791	2.151	24	24	0.033
E2	Single measure	0.064	-0.352	0.449	1.132	24	24	0.382
	Average measure	0.121	-1.086	0.620	1.132	24	24	0.382
E3	Single measure	-0.193	-0.566	0.226	0.689	24	24	0.817
	Average measure	-0.478	-2.612	0.369	0.689	24	24	0.817
E4	Single measure	0.171	-0.250	0.530	1.396	24	24	0.210
	Average measure	0.292	-0.667	0.693	1.396	24	24	0.210

E1: Examiner 1; E2: Examiner 2; E3: Examiner 3; E4: Examiner 4; NHP: Natural head position; OP: Oriented position.

The data was compared individually for each examiner between times T1 and T2. Examiner 1, represented by an orthodontist, showed reasonable agreement for both NHP and OP. Examiners 2 (orthodontist), 3 (oral and maxillofacial surgeon), and 4 (professional photographer) showed poor agreement. Thus, only examiner 1 showed that he was calibrated to take photographs for both NHP and OP. The other examiners (2 and 3), although dental surgeons, were not calibrated, and finally, examiner 4 (professional photographer) had no training in the health area. 

**Figure 2:**
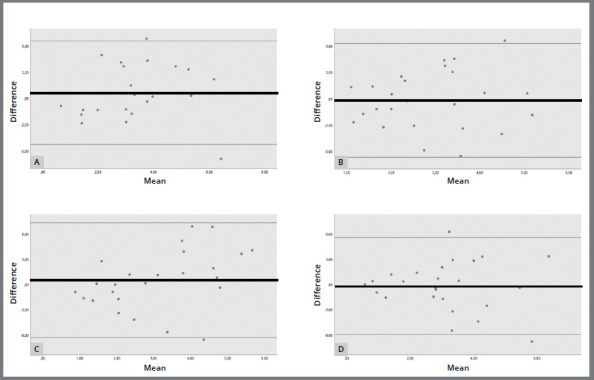
Bland-Altman diagram of the comparison between NHP and OP at T1 for each examiner: A) Examiner 1, B) Examiner 2, C) Examiner 3 and D) Examiner 4. Black line: mean of the differences in measurements. Gray lines: 95% interval of the distributions of the measured differences.

**Figure 3:**
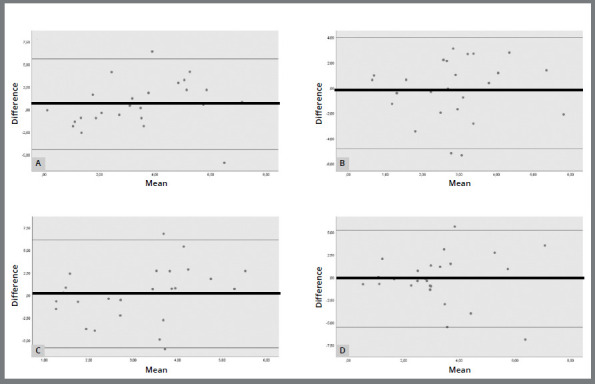
Bland-Altman diagram of the comparison between NHP and OP in T2 for each examiner: A) Examiner 1, B) Examiner 2, C) Examiner 3 and D) Examiner 4. Black line: mean of the differences in the measurements. Gray lines: 95% interval of the distributions of the measured differences.

The graphs resulting from the Bland-Altman analysis (Figs 2 and 3) were used to assess the agreement between NHP and OP for each examiner at T1 and T2. Both techniques were in agreement, but the graphs did not show any difference between the techniques in terms of reproducibility. 

There was agreement between the NHP and OP methods when different examiners performed the photographic recordings, both for T1 and T2. Overall, no mean difference was greater than 1°, and these means were also not statistically significant in relation to zero (p>0.05), and most of the values were within the 95% confidence interval. 

## DISCUSSION

Clinical photographs play an important role in orthodontic, orthopedic and surgical treatments; they are relevant in the initial documentation for diagnosis and planning, comparison during and after treatment, allowing long-term follow-up of the clinical case, facilitating communication between professionals and also between the professional and the patient.^5^ They are also used as court documents, marketing, teaching, and learning.^3^ They therefore need to be reliable, accurate, standardized, and reproducible.^5^

The NHP has been extensively researched in several studies and has been proven to be a reliable and standardized method.^8^ ^,^ ^9^ ^,^ ^11^ ^,^ ^16^ ^,^ ^18^ Its reproducibility has been confirmed by several studies.^10^ ^,^ ^12^ ^,^ ^25^ ^,^ ^26^ During photographic recordings, some patients may adopt an erroneous NHP that does not match their real condition. Physiological, psychological and pathological factors are related to the natural posture of the head.^18^ ^,^ ^26^ In these circumstances, it is necessary to position the patient’s head according to the professional’s perspective. OP emerges not as a new position, but from the need to correct the position of the patient’s head in cases where it is flexed, extended or out of correct anatomical alignment; therefore, the professional must know the technique and training.^14^

The variability in the standardization of head orientation between different examiners has been investigated.^27^ The results suggest that the standardization of anatomical alignment in OP can be difficult to achieve, since the perception of correct head alignment varies over time and differs between observers.^27^ Special care should be taken so that the professional does not underestimate cases of skeletal malocclusion, reducing the severity of the deformity, especially in skeletal Class III patients.^17^ The recording of photographs in NHP and OP in profile views in this study was conducted because only in this plane the sagittal relationship of the mandible to the cranial base can be evaluated, allowing for the correct classification of facial pattern alterations.

In the present study, inter-examiner reproducibility was observed in T1 (ICC = 0.706 for NHP and ICC = 0.708 for OP) and T2 (ICC = 0.739 for NHP and ICC = 0.704 for OP), but not intra-examiner reproducibility. Only examiner 1 showed reasonable reliability (ICC = 0.504 for NHP and ICC = 0.530 for OP). The experience of the professionals taking the photographs can directly influence the anatomical alignment of the head, resulting in less variation and greater constancy of the OP.^28^ The different levels of experience, training, and specialization among the examiners were reflected in the present results. The training and calibration of the professional is a relevant factor,^3^ ^,^ ^5^ ^,^ ^29^ as well as: a suitable environment, equipment, storage of the images, in order to keep them standardized and with quality.^3^ Capon^29^ highlighted that other factors can directly interfere in the photographic recordings, such as patient compliance and collaboration during the recordings, the photographer’s ability to interpret the correct alignment of the head, the patient’s age (greater difficulty in children) and adults with learning difficulties. Questionnaires showed that 30% of clinical photographers were not familiar with NHP, and only 12% of those interviewed (2 orthodontists and 1 oral and maxillofacial surgeon) justified NHP as a reproducible position that is closer to the individual’s reality. The use of Frankfort plane as a reference for head alignment was reported by 77% of the participants. This is contrary to new trends in photographic records, given the great individual variability of craniofacial reference lines in relation to the true vertical and horizontal lines.^8^ ^,^ ^9^ ^,^ ^11^ ^,^ ^12^ ^,^ ^25^

Although NHP has been known and introduced in the literature since the 1950s, there is a lack of knowledge among professionals who work with clinical photographs. A wider dissemination of the technique is suggested, since it allows for a more reliable photographic image of the patient to be reproduced, as well as being reproducible over time. 

In clinical practice, it has been found that most of the errors in photographs are related to the patient’s head and the positioning of the chin.^5^ The anterior contour of the mandibular symphysis, represented by OP, suffers a change in its sagittal position in relation to the true vertical line when the head is rotated upwards or downwards.^6^ The difficulty in rotating the head in the vertical direction can also be explained by the lack of facial references in this axis; when analyzing a face three-dimensionally, the eyes, ears, nose, and facial contour are “insights” that help us to correctly align the head anatomically - while in the sagittal direction these parameters are not present.^27^

The guidelines followed in this study for recording the NHP were those described by Solow and Tallgren^11^ and used by other authors.^4^ ^,^ ^6^ ^,^ ^7^ ^,^ ^8^ ^,^ ^10^ ^,^ ^15^ ^,^ ^16^ ^,^ ^17^ ^,^ ^25^

The present study was conducted with standardized distances between the individual, the mirror, and the tripod with the professional camera, in addition to the 100mm macro lens, as also used by Tôrres et al.^6^ The 28mm or 55mm lenses are inadequate when compared to the 100mm macro lenses, which allow proper facial framing and keep all the images within the real size of the individuals, with precision and maintenance of characteristics and deviations.^19^ It is recommended that the places that take the photographs should have more assertive standardization in relation to the position of the head, both in the technique and in the use of the equipment. The lack of standardization is a reality in the clinical routine, resulting in unreliable images that do not allow a comparative assessment of before, during and after treatment, as well as an assessment of facial growth.^30^

In order to identify the true vertical line in the photographic records, together with the patient’s face, some researchers have used a chain with a lead plummet.^6^ ^,^ ^11^ ^,^ ^14^ ^,^ ^16^ In this study, the true vertical and horizontal lines were represented using a checkered background fixed to the wall in the background, similar to Bjerin^8^ and Lundström et al.^18^, who used a horizontal line drawn on the wall behind the individual. 

To avoid discrepancies when marking the facial points, calibration was carried out by measuring the angles of all the photographs at two different times and comparing the mean angles obtained: it was observed that they differed by less than 1 degree, proving the researcher’s calibration. A study by Fattahi et al.^12^ also adopted the researcher’s calibration in a similar way. The angles measured in this study were taken individually and digitally with automatic calculation, obtaining more precise measurements, when compared to studies that took manual measurements. 

Regarding sample size, this study initially had 30 individuals, similar to the study by Lundström et al^18^ (28 individuals) and Lundström et al^14^ (27 individuals). However, during the course of the study, five individuals were excluded because their perioral muscles were not relaxed when the photographs were taken.^11^ Therefore, the final sample consisted of 25 individuals, resulting in a 95% confidence interval and a margin of error of 8.14%, which is considered acceptable. Further studies with a larger and more diverse sample could be of great importance in assessing the reliability of facial measurements in different populations and clinical contexts. 

## CONCLUSIONS

The reproducibility of the photographic records of the facial profile for both NHP and OP showed good inter-examiner reliability at times T1 and T2. However, intra-examiner reliability was variable at both times, being reasonable only for examiner 1 (orthodontist), suggesting that consistency within the same examiner may be influenced by technical or training factors.

Bland-Altman’s analysis confirmed good agreement between the two positioning techniques, NHP and OP, which validate the use of both in photographic facial profile studies. This suggests that both NHP and OP can be used reliably in research and clinical practice, provided they are carried out by properly trained professionals. In short, the facial profile photography technique in the two positions showed adequate reproducibility and is a useful tool in the aesthetic and functional assessment of the facial profile.
